# Muscle Loss in Chronic Liver Diseases: The Example of Nonalcoholic Liver Disease

**DOI:** 10.3390/nu10091195

**Published:** 2018-09-01

**Authors:** Jean-Pascal De Bandt, Prasanthi Jegatheesan, Naouel Tennoune-El-Hafaia

**Affiliations:** EA4466 PRETRAM, Faculté de Pharmacie de Paris, USPC, 75006 Paris, France; pira_jegatheesan@hotmail.com (P.J.); naouel.el-hafaia@parisdescartes.fr (N.T.-E.-H.)

**Keywords:** sarcopenia, protein metabolism, insulin resistance, endoplasmic reticulum stress, hepatokine, amino acid, uric acid, methylglyoxal

## Abstract

Recent publications highlight a frequent loss of muscle mass in chronic liver diseases, including nonalcoholic fatty liver disease (NAFLD), and its association with a poorer prognosis. In NAFLD, given the role of muscle in energy metabolism, muscle loss promotes disease progression. However, liver damage may be directly responsible of this muscle loss. Indeed, muscle homeostasis depends on the balance between peripheral availability and action of anabolic effectors and catabolic signals. Moreover, insulin resistance of protein metabolism only partially explains muscle loss during NAFLD. Interestingly, some data indicate specific alterations in the liver–muscle axis, particularly in situations such as excess fructose/sucrose consumption, associated with increased hepatic de novo lipogenesis (DNL) and endoplasmic reticulum stress. In this context, the liver will be responsible for a decrease in the peripheral availability of anabolic factors such as hormones and amino acids, and for the production of catabolic effectors such as various hepatokines, methylglyoxal, and uric acid. A better understanding of these liver–muscle interactions could open new therapeutic opportunities for the management of NAFLD patients.

## 1. Introduction

The loss in muscle mass, often wrongly referred to as sarcopenia, has long been largely neglected in liver diseases. This is probably partly because of possible difficulties in its assessment due, for example, to fluid retention. However, several recent studies have shown frequent loss of muscle mass associated with poor prognosis in various chronic liver diseases. The progressive deterioration of muscle trophicity in these diseases is therefore receiving increasing attention. Overall, studies demonstrate muscle loss in nearly 60% of patients with end-stage liver diseases and this is associated with a worse prognosis [1]. However, muscle loss is already present in the early stages of liver disease and worsens with its severity [2]. This is particularly the case of nonalcoholic fatty liver disease (NAFLD), which encompasses a broad spectrum of disorders ranging from simple steatosis to nonalcoholic steatohepatitis (NASH), cirrhosis, and hepatocellular carcinoma, and where loss of muscle mass can occur very early during the disease. Indeed, in addition to the disturbances in muscle homeostasis related to metabolic disorders, several pieces of evidence indicate a specific role of the alterations in liver function in muscle loss. This is particularly important as the progressive deterioration of muscle trophicity promotes NAFLD progression, given the role of the muscle in energy metabolism. The alterations in the liver–muscle axis, due to hepatic steatosis, initiate a vicious circle in which liver disease favors defective muscle protein accretion and in which muscle loss favors metabolic alterations as well as hepatic steatosis and inflammation.

After examining some epidemiological data on muscle loss in chronic liver diseases and NAFLD, the authors of this review focus on muscle homeostasis and the different mechanisms by which NAFLD can act on muscle protein metabolism, including the decreased peripheral availability and action of anabolic factors such as hormones and amino acids, and the production of catabolic effectors such as various hepatokines, methylglyoxal, and uric acid.

It should be noted that many authors speak of sarcopenia while patients present only this loss of muscle mass. Indeed, the European society for clinical nutrition and metabolism defines sarcopenia, a term first coined for geriatric patients, as “a syndrome of its own characterized by the progressive and generalized loss of skeletal muscle mass, strength and function (performance) with a consequent risk of adverse outcomes” [3].

## 2. Muscle Loss in Chronic Liver Diseases

The interest in muscle mass loss in liver diseases is the result of recent evidence of its frequency and its consequences in terms of morbidity and mortality. A meta-analysis of 7 Asian studies and 13 Western studies in patients with liver cirrhosis of various etiologies concluded that muscle loss was present in 48.1% of patients, more prevalent in men (61.6%) than in women (36%) [4]. Several studies have shown that muscle loss in liver transplant candidates is associated with increased mortality [2] and increased risk of complications [4] In a recent systematic review and meta-analysis, the pooled hazard ratios of muscle loss was 1.84 (95% CI: 1.11–3.05, *p* = 0.02) for post-transplantation mortality [5]. Moreover, muscle loss appeared to be associated with increased complications, such as infections [6], ascites, encephalopathy, and variceal hemorrhage [7].

More specifically for NAFLD, most data come from studies in the Korean population. Using data from the Korean National Health and Nutrition Examination Surveys (KNHANES), Lee et al. [8] showed that NAFLD was present in 2761 (28.5%) out of 9676 subjects, and that 337 NAFLD patients (12.2%) had low muscle mass. Hong et al. [9] studied the relationship between muscle mass and liver disease evaluated by serum gamma-glutamyl transferase (GGT) in 3193 adults aged over 50 years from the fifth KNHANES. They observed that patients in the highest GGT quintile were 2.3 times more likely to have low muscle mass than those in the lowest quintile. In a seven-year Korean longitudinal study [10] on 12,624 subjects without initial NAFLD, followed for occupational medicine purpose, 14.8% of the subjects developed NAFLD and the highest tertile of muscle mass was inversely associated with the incidence of NAFLD compared to the lowest tertile. Finally, in the study by Koo et al. [11] of 309 subjects with signs of hepatic steatosis, the prevalence of low muscle mass was 8.7% in patients without NAFLD and 17.9% and 35%, respectively, in biopsy-proven nonalcoholic fatty liver and NASH patients. In this study, as in Lee’s study [8], low muscle mass was associated with fibrosis (OR: 2.05, 95% CI: 1.01–4.16, *p* = 0.034), independently of body mass index and insulin resistance. This relationship between low muscle mass and fibrosis has also been confirmed in a European cohort of biopsy-proven NAFLD patients [12].

Given the importance of muscle in energy and nitrogen homeostasis, this muscle loss negatively affects whole-body metabolism [13]. The consequences on hepatic metabolism are clearly shown, for example, in the study by Flannery et al. [14] of healthy sedentary elderly subjects. These authors observed, after a test meal, a twofold higher hepatic de novo lipogenesis (DNL), a threefold higher postprandial hepatic triglyceride (TG) content, and significantly increased plasma TG compared to healthy, young subjects. Note that, in a study of 452 apparently healthy adults from the Korean Sarcopenic Obesity Study [15], patients in the lowest quartile of muscle mass had a 5.2-fold increased risk of NAFLD compared to the highest quartile (95% CI: 1.63–16.33, *p* = 0.041). In this study, hepatic steatosis and skeletal muscle mass index were negatively correlated with insulin resistance, low grade inflammation, and arterial stiffness, and positively correlated with plasma TG and alanine aminotransferase.

## 3. Muscle Protein Homeostasis

To understand the mechanisms by which hepatic disorders may affect muscle protein homeostasis, the authors will first briefly review some main aspects of the control of protein metabolism in muscle. Muscle homeostasis in healthy individuals depends on the fasting–feeding alternation and, at the cell level, on the balance between protein synthesis and catabolism. Protein synthesis is activated by anabolic factors such as hormones (insulin and insulin-like growth factor 1 [IGF-1]), and amino acid (AAs) availability (with AAs such as leucine and arginine playing specific regulatory roles), and it is inhibited by nutrient deficiency and inflammatory processes. Conversely, protein catabolism is activated by energy and AA deficiency and inflammatory processes, and it is inhibited by anabolic hormones [16]. 

During the postprandial period, anabolic effects of feeding result from increased protein synthesis and decreased catabolism due to increased availability and action of anabolic effectors. AAs stimulate protein synthesis; insulin has a permissive effect and increases the supply of nutrients to muscles through its vasodilatory properties. Insulin-induced inhibition of proteolysis is enhanced by AA availability [17]. In addition, the activation of protein synthesis depends on specific AAs such as leucine and arginine; thus, the anabolic effects of feeding also depend on specific qualitative variations in AA availability [18]. Finally, the vasodilatory effects of insulin play an important role in the physiological coupling between hemodynamic and metabolic homeostasis [19]. The increase in the metabolic activity of skeletal muscle requires adequate availability of the substrate for nitric oxide (°NO) synthesis (i.e., arginine) to increase blood flow for substrate supply.

On the long term, maintaining skeletal muscle mass also depends on the growth hormone (GH)/IGF-1 axis. Blood IGF-I is 80% dependent on its GH-induced liver production. Plasma IGF-I is associated positively with lean body mass and muscle function, and negatively with body fat mass. IGF-I action is also regulated by its binding to circulating IGF-1 binding proteins (IGFBP) produced by the liver [20].

Overall, the maintenance of muscle mass requires that the muscles be responsive to these anabolic effectors and that the anabolic effectors actually and quantitatively reach the muscles. As indicated below, NAFLD may be associated with a decrease in muscle mass as a consequence of (i) insulin-resistance, (ii) decreased IGF-1 production by the liver, (iii) decreased AA flows to the muscles due to increased splanchnic utilization, (iv) a defective postprandial peripheral vasodilatory response, or (v) the catabolic effect of various mediators, including hepatokines, produced by the liver in steatosis-induced situations of endoplasmic reticulum (ER) stress. 

## 4. Insulin Resistance and Muscle Homeostasis

A first process that can promote muscle loss is insulin resistance. Depending on the pathophysiological situation, insulin resistance may either precede NAFLD, due to increased adiposity, or result from NAFLD as the steatotic liver is the site of increased glucose and TG production; in all cases, the two processes reinforce each other. Decreased sensitivity of protein turnover to insulin action has been described in both type 1 and type 2 diabetes [21]. This decrease in insulin sensitivity may result from several mechanisms such as lipotoxicity, glucotoxicity, and inflammation.

Increased circulating free fatty acid (FFA) concentration favors ectopic lipid deposition and altered insulin signaling due to the accumulation of TG deposition, for example, in muscle [22]. Moreover, specific FFAs (e.g., palmitate) and lipid metabolites (e.g., ceramide and diacylglycerol) may induce insulin resistance. It should be noted that palmitate also activates toll-like receptor 4 (TLR4) and triggers inflammatory processes [23].

Excess glucose induces oxidative stress in muscles and the production of advanced glycation end products (AGEs) that interact with the receptors of AGEs on muscle cells. These receptors induce the production of reactive oxygen species and cause inflammation via mitogen-activated protein kinases and nuclear factor κB pathways [24]. For example, Howard et al. [25] showed that high glucose and AGEs induce a defect in myocyte membrane repair. 

Excess adipose tissue, particularly visceral adipose tissue, is associated with the increased secretion of pro-inflammatory cytokines, such as tumor necrosis factor α and interleukin-6, and adipokines (leptin and resistin, whereas insulin-sensitizing adiponectin production decreases) which promote insulin resistance [26]. Interestingly, in the authors’ model of fructose-induced NAFLD in rats, muscle loss was associated with increased visceral adiposity and an inflammatory state [27].

A last mechanism by which insulin resistance may contribute to muscle loss is the above-mentioned defect in the postprandial peripheral vasodilatory response as it reduces the flow of anabolic factors to the muscle. Indeed, in addition to its essential metabolic actions, insulin stimulates the production of nitric oxide (°NO) by endothelial °NO synthase (eNOS). Therefore, in the post-prandial period, insulin induces vasodilation, increased blood flow and increased availability of substrates and hormones for target tissues. Insulin resistance is associated with impaired endothelium-dependent vasodilation and vascular function [19]. There is thus a reciprocal interaction between insulin resistance and the defect in endothelial °NO production. The pathophysiological mechanisms linking these two processes contribute to metabolic disorders and the cardiovascular features of the metabolic syndrome. Moreover, insulin resistance states are associated with decreased availability of an important AA, arginine, the substrate of eNOS for °NO synthesis.

## 5. Metabolic Disorders, Liver Steatosis, and Endoplasmic Reticulum Stress

Although insulin resistance may affect muscle function, it cannot by itself explain the defect in muscle protein accretion associated with NAFLD and, more specifically, with the excessive consumption of fructose. Therefore, another process must contribute to muscle protein loss. One explanation could be excess DNL, the cause of steatosis, leading to hepatic oxidative stress, inflammation, and ER stress.

Under normal conditions, at the hepatic level, FFAs come from white adipose tissue (WAT) lipolysis during the fasting period, from diet, and from DNL. FFAs will either be esterified into TG and then exported to the blood as very-low-density lipoproteins (VLDL) or degraded by β-oxidation. Pathological accumulation of lipids in the liver may result from the excessive entry of FFAs released by insulin-resistant WAT, particularly visceral adipose tissue, excessive activation of DNL, and alterations in β-oxidation and lipid excretion as VLDL. In NAFLD patients, approximately 60% of FFAs come from lipolysis in WAT, nearly 25% result from DNL, and the remainder come from diet [28]. Nutritional factors, such as a diet rich in sucrose/fructose and lipids, and the disequilibrium of the energy balance thus play a major role in the occurrence of this pathology.

Excessive sucrose or fructose consumption can significantly contribute to liver steatosis and disease progression. Experimental studies repeatedly demonstrated that a high-sucrose or high-fructose diet promotes subcutaneous and visceral obesity, insulin resistance, dyslipidemia, increased blood uric acid, and hypertension [29]. Even moderate doses of fructose can induce metabolic syndrome, fatty liver, and type 2 diabetes even in the absence of excess energy intake [30]. In humans, a high-fructose intake (>1.5 g/kg/day) can double the intrahepatic fat content within six days [31]. Conversely, in a pilot study of 15 NAFLD patients, Volynets et al. [32] showed that a dietary intervention focusing on a 50% reduction in fructose intake was associated with decreased hepatic steatosis and improved liver function and glucose tolerance. This can be at least in part explained by the specificities of fructose metabolism in the liver and the excessive activation of DNL. Indeed, fructose is both a substrate and an activator of DNL through the activation of carbohydrate-responsive element-binding protein (ChREBP) and sterol regulatory element-binding protein 1c, two transcription factors controlling the main enzymes involved in DNL [33]. Excess DNL causes oxidative stress in liver cells and is associated with the development of hepatic inflammation and insulin resistance [34,35]. It also induces ER stress, which contributes to the aggravation of NAFLD [34]. The superposition of ER stress and inflammation may lead to the production of various mediators, such as cytokines, hepatokines, and carbohydrate and lipid derivatives, which can act at the whole-body level and contribute to alterations in whole-body metabolism [36,37].

With respect to muscle mass loss, studies [27,38,39] have shown that excessive consumption of fructose or sucrose leads to changes in body composition with fat accumulation and alterations of muscle protein pool. Interestingly, in a study of community-dwelling elderly subjects, Laclaustra et al. [40] showed an association between added sugar consumption and the appearance of frailty. Several lines of evidence indicate that sucrose-related alterations in liver metabolism, at least in part due to the conversion of sucrose into lipids in the process of DNL, lead to changes in peripheral organ metabolism and muscle protein accretion. This is illustrated by the comparison of mouse models of primary steatosis, resulting from an increase in hepatic DNL, and secondary steatosis resulting from ectopic lipid deposition, primary steatosis being associated with significantly lower lean body mass [41].

## 6. Hepatic Endoplasmic Reticulum Stress and Muscle Homeostasis

The question therefore arises about the mechanisms by which these alterations in liver homeostasis may affect muscle protein metabolism. 

### 6.1. Alterations of the GH/IGF-1 Axis

The bidirectional relationship between steatosis and insulin resistance has already been mentioned. Steatosis is also associated with alterations of the GH/IGF-1 axis. While data on plasma GH are inconsistent [42,43], studies agree on a decrease in plasma IGF-1 in NAFLD [42,43,44]. The liver being the main organ contributing to plasma IGF-1 concentration, this suggests that NAFLD alters IGF-1 production. Runchey et al. [45] showed in 4172 adults who participated in the NHANES III that the highest quartiles of IGF-1 and IGF-1/IGFBP-3 were associated with a lower likelihood and grade of NAFLD. Note that in an exploratory study of healthy volunteers on a high-sucrose diet, the authors observed an association between increased liver lipid content and decreased plasma IGF-1 [46]. In a model of Western diet-induced NAFLD in mice, steatosis was associated with a 40% decrease in IGF-1 hepatic expression [47]. This seems to be specific for liver steatosis, as suggested by Chishima et al. [43] in a study of Japanese patients with NAFLD or Hepatitis C virus (HCV) chronic liver disease where IGF-1 levels were decreased in patients with NAFLD but not with HCV. Moreover, serum IGFBP-3 levels were also decreased, indicating a reduction in blood half-life of IGF-1. Conversely, hepatic ER stress is associated with the stimulation of IGFBP1 secretion [48]. IGFBP1 is a modulator of IGF-1 action and its overexpression is associated with hyperinsulinemia and glucose intolerance [49]. Experimentally, hepatic steatosis-related alterations in GH/IGF-1 axis are associated with a decrease in muscle myofibrillar protein content and a reduction in muscle strength in the absence of significant inflammation [47].

### 6.2. Alterations of Amino Acid Interorgan Fluxes

Disorders of nitrogen homeostasis in situations of stimulated DNL may be an early event during the development of steatosis [33]. In an experimental model of fructose-induced NAFLD in rats, the prolonged administration of a fructose-rich diet was associated with a decrease in lean body mass, an increase in visceral fat mass, and changes in AA plasma levels, notably in arginine bioavailability. Conversely, an increase in AA availability enable to decrease DNL and associated alterations in body composition [27]. In healthy volunteers, an essential AA supplement reduced fructose-induced intrahepatic lipid accumulation [50]. Interestingly, based on their results showing alterations in blood AA profile in non-diabetic NAFLD patients with or without obesity, Gaggini et al. [51] concluded that the observed increase in AAs such as branched-chain and aromatic AAs resulted from an increase in muscle proteolysis. Finally, in overweight hypertriglyceridemic patients, fructose infusion was associated with altered AA plasma levels and increased splanchnic extraction [33]. Similarly, postprandial AA availability was impaired in healthy volunteers receiving a high-sucrose diet [46]. Taken together, these data suggest that excess hepatic DNL is associated with a reorientation of AA fluxes towards the liver at the expense of muscle protein homeostasis.

### 6.3. Hepatokines and Muscle Homeostasis

In situations of hepatic ER stress, induced for example by steatosis, an increased production of some hepatokines and inflammatory cytokines has been demonstrated [52]. In cultured hepatocytes, steatosis has been associated with changes in the secretion of approximately 30 hepatokines [53]. Some of these mediators may be involved in muscle loss through a direct effect or through the deterioration of insulin sensitivity. For example:

(1) Fetuin A is a glycoprotein associated with alterations in glucose and lipid metabolism. Both experimental and clinical studies showed increased hepatic expression and plasma levels of Fetuin A during NAFLD. Studies in humans showed a close relationship between plasma Fetuin A and the metabolic syndrome [54]. Its production by hepatocytes is strongly stimulated by ER stress [55]. This protein produced primarily by the liver is an endogenous inhibitor of the insulin receptor tyrosine kinase in skeletal muscle [56]. Fetuin A may also bind TLR4, stimulating inflammatory pathways [52].
(2) Fibroblast growth factor 21 (FGF21) is a mediator produced primarily by the liver that contributes to the regulation of energy metabolism and insulin sensitivity [57]. FGF21 is now recognized as a key player in the adaptive response to starvation and feeding [58]. Fructose induces FGF21 production by the activation of the transcription factor ChREBP [59]. ER stress modulates FGF21 expression in the liver [60]. FGF21 is an intriguing hepatokine as it is generally considered as beneficial [59]; however, some data indicate either states of FGF21 resistance or deleterious effects, as suggested by the very high FGF21 plasma levels observed in patients with insulin resistance [52]. In male and female rats fed a high-fat high-fructose diet, FGF21 was increased only in males and this was associated with marked liver damage, inflammation, and oxidative stress [61]. AA deprivation is also a potent inducer of hepatic FGF21 production via ER stress response [62]; very high levels of FGF21 could potentially alter nitrogen homeostasis in the context of NAFLD-associated muscle mass loss. It should be noted that transgenic mice overexpressing FGF21 exhibit increased gluconeogenesis in the fed state, and that acute treatment with FGF21 induces key enzymes in the gluconeogenic pathway [62], suggesting an increased hepatic utilization of AAs.
(3) Hepassocin (HPS, hepatocyte-derived fibrinogen-related protein 1 [HFREP1], fibrinogen-like protein 1 [FGL1]) is increased in NAFLD and induces insulin resistance in muscle [52]. Wu et al. have shown in humans that increased plasma HPS is independently associated with insulin resistance [63]. In primary hepatocytes or in vivo in mice, HPS expression is induced by ER stress. In differentiated myotubes, depending on the dose, HPS activated the c-Jun N-terminal kinase inflammatory pathway and altered insulin sensitivity [64]. In mice, hepatic HPS overexpression induced insulin resistance, while its knockdown was associated with improved insulin sensitivity in high-fat fed mice [63].

### 6.4. Hepatic Production of Catabolic Factors

Finally, carbohydrate metabolism may lead to the release by the liver of metabolites with significant peripheral effects.

During normal glycolysis, small amounts of methylglyoxal (MG) may be released. MG is produced by the fragmentation of the two products of aldolase B, namely glyceraldehyde-3-phosphate and dihydroxyacetone phosphate. MG is a powerful glycating agent leading to the generation of AGEs. Under normal conditions, its formation rate represents 0.1–0.4% of glycolytic flux [65], but the acceleration of glycolytic flux by fructose greatly increases MG formation. MG induces endothelial dysfunction [66]. Dhar et al. [67] showed an increase in MG formation associated with hypertension in fructose-fed rats. By acting on vascular function, MG may therefore contribute to a decrease in the peripheral availability of anabolic hormones and AAs.

A high-fructose intake results in an increased plasma uric acid level [39,68]. First, uric acid can contribute to fructose-induced metabolic disorders by impairing endothelial function, thereby decreasing insulin sensitivity by preventing insulin-induced muscle vasodilation. In addition, Zhu et al. [69] recently showed in mice that uric acid inhibits insulin signaling in muscle and induces insulin resistance. This effect may be related to uric acid-induced oxidative stress. In a study of severely obese subjects, Fabbrini et al. [70] observed a 40% decrease in insulin sensitivity in those with increased plasma uric acid. In an analysis of data from 7544 subjects who participated in the NHANES III, Beavers et al. [71] showed an association between increased blood uric acid levels and low muscle mass.

## 7. Conclusions

Chronic liver diseases contribute to alterations in muscle protein homeostasis, already at the stage of hepatic steatosis. While this review is limited to alterations in the liver–muscle axis, the situation is probably even more complex because NAFLD can be considered as a systemic disease affecting not only the liver and muscles, but also the gut and adipose tissue. High-fat and high-fructose diets, which promote the development of NAFLD, are associated with alterations in gut microbiota, increased gut permeability, and bacterial toxin translocation that can affect muscle homeostasis through systemic inflammation and insulin resistance [72]. Similarly, excessive sucrose consumption is associated with an increase in visceral adipose tissue in which has a higher production of pro-inflammatory factors and metabolic alterations than subcutaneous adipose tissue [26]. Muscle mass and function can be influenced by this adipose tissue dysfunction. The difficulty now becomes to establish the respective contribution of these different mechanisms in order to better define therapeutic targets.

## Abbreviations

AA	amino acid
AGE	advanced glycation end products
ChREBP	carbohydrate-responsive element-binding protein
DNL	de novo lipogenesis
eNOS	endothelial °NO synthase
ER	endoplasmic reticulum
FFA	free fatty acid
FGF21	fibroblast growth factor 21
GGT	gamma-glutamyl transferase
GH	growth hormone
HCV	Hepatitis C virus
HPS	hepassocin
IGF	insulin-like growth factor
IGFBP	IGF binding protein
KNHANES	Korean National Health and Nutrition Examination Surveys
MG	methylglyoxal
NAFLD	nonalcoholic fatty liver disease
NASH	nonalcoholic steatohepatitis
NHANES	national health and nutrition examination survey
°NO	nitric oxide
TG	triglycerides
TLR4	toll-like receptor 4
VLDL	very-low-density lipoprotein
WAT	white adipose tissue
